# Supplementary material for: Rapid discovery of high-affinity antibodies via massively parallel sequencing, ribosome display and affinity screening
Source: Nat Biomed Eng. 2023 Oct 9;8(3):214–32. doi: 10.1038/s41551-023-01093-3 (PMC10963267; doi:10.1038/s41551-023-01093-3)
Supplement: Supplementary file 1 — Supplementary Figs. 1–14 and Tables 1–14. [file 41551_2023_1093_MOESM1_ESM.pdf]

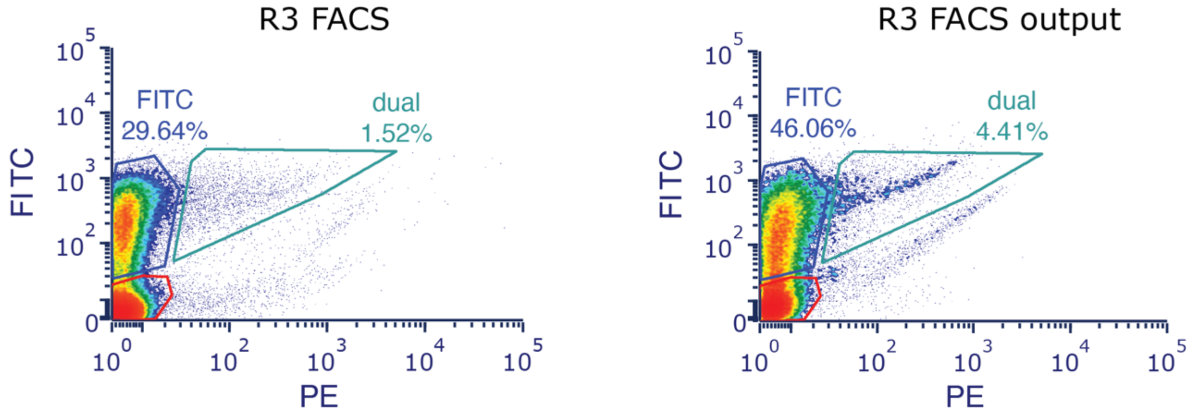

Supplementary Fig. 1 l Flow cytometry plots from the nanobody Round 3 FACS showing cells expressing nanobody with a FITC-anti-HA antibody and cells binding to HEL-biotin-Streptavidin-PE. The left panel shows the dual gating that sorted 1.52% of all events and the right panel shows enrichment in the dual gate after recovery from sorting.

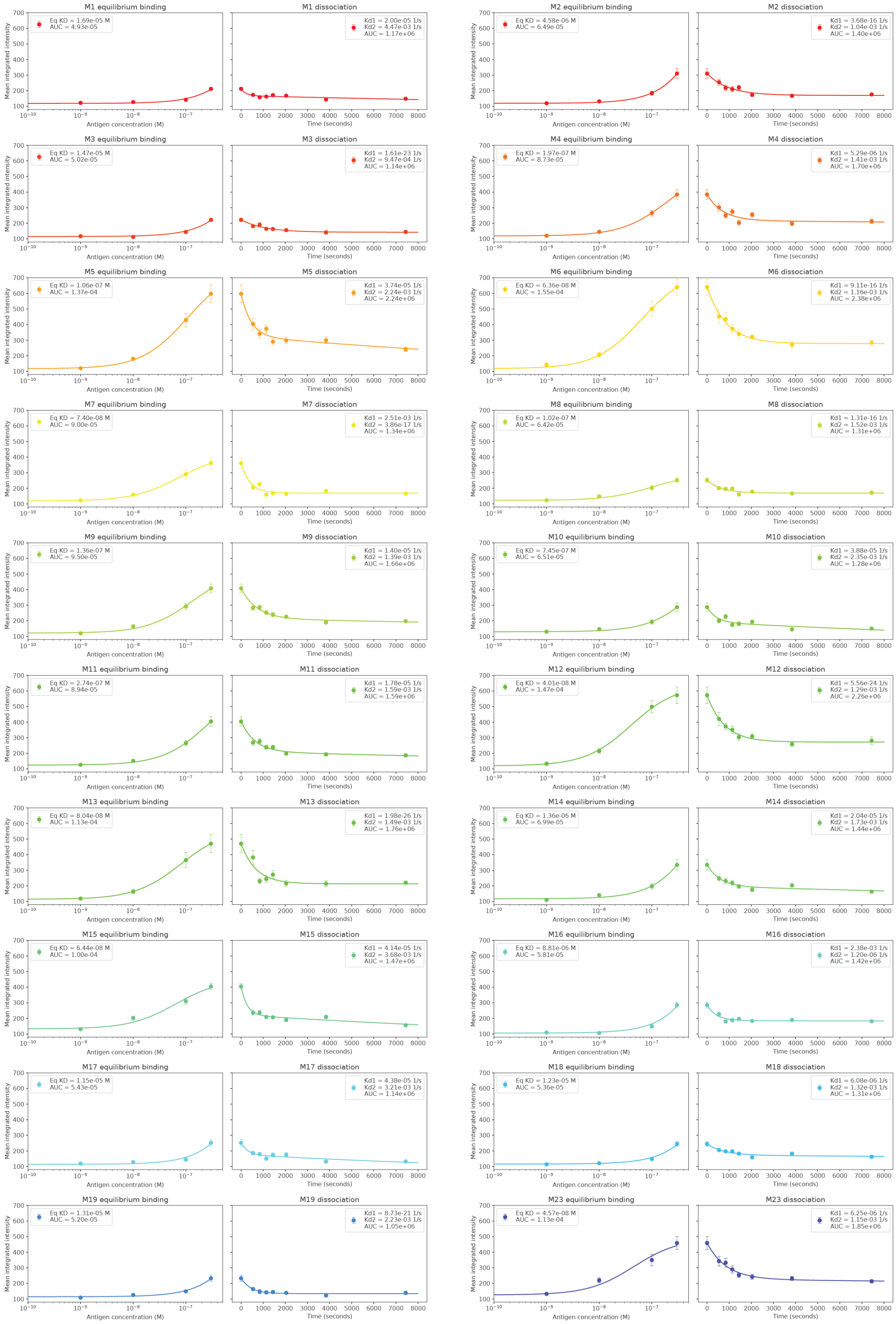

Supplementary Fig. 2 | Deep screening derived equilibrium binding and kinetic dissociation curves for the anti-HEL nanobody clones selected from the MACS library for characterisation. Each concentration condition within curve represents at least 12 measurements from a deep screening experiment. Error bars are SEM and n≥12 technical replicates of a given UMI. We report an equilibrium KD, area under the curve (AUC) for the equilibrium binding and two dissociation rates for a biphasic dissociation model, as well as an AUC.

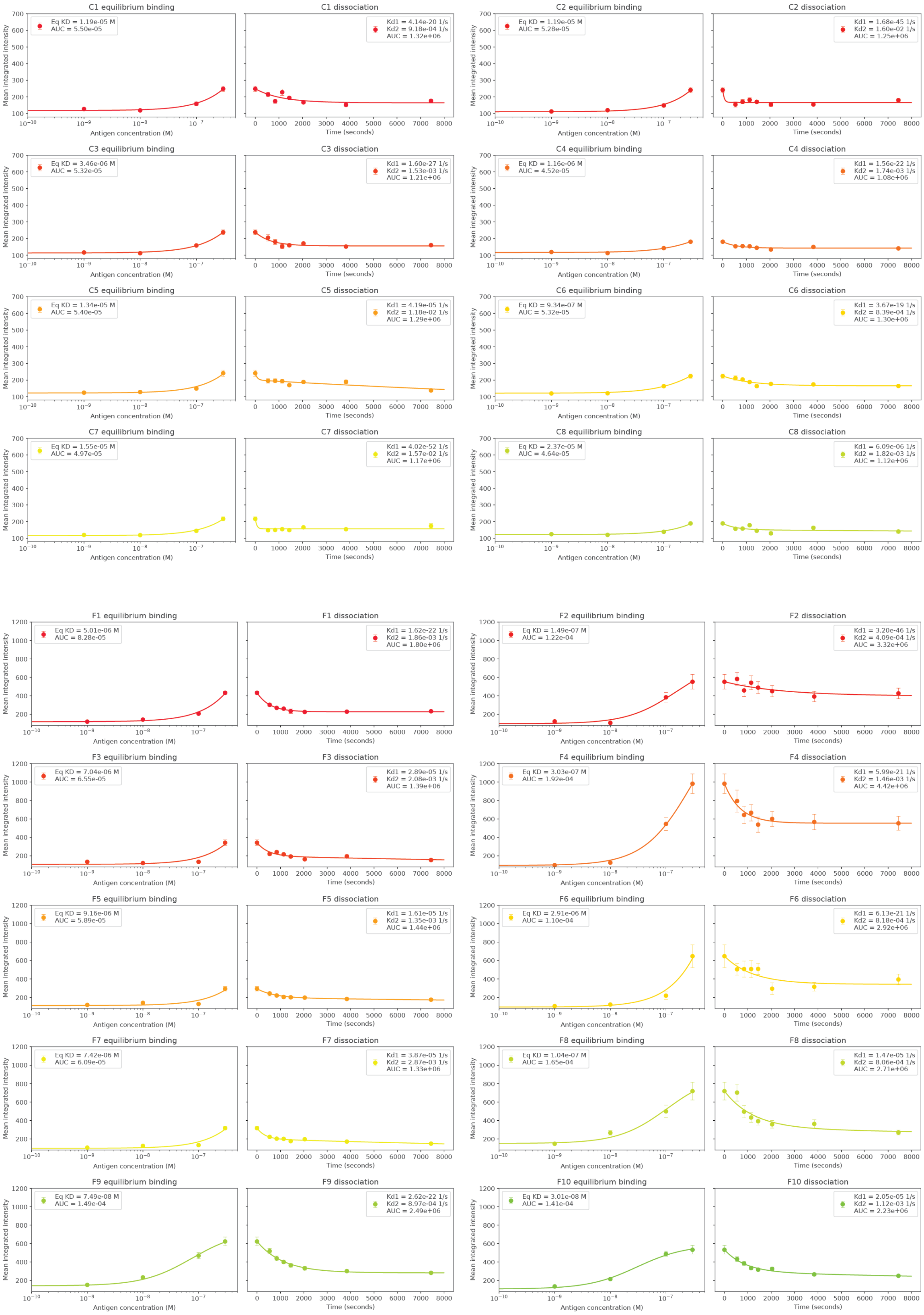

Supplementary Fig. 3 I Deep screening derived equilibrium binding and kinetic dissociation curves for the anti-HEL nanobody clones selected by picking 96 colonies from the R3 MACS output and clones selected from the R3 FACS library screen. Each concentration condition within curve represents at least 12 measurements from a deep screening experiment. Error bars are SEM and n≥12 technical replicates of a given UMI. We report an equilibrium KD, area under the curve (AUC) for the equilibrium binding and two dissociation rates for a biphasic dissociation model, as well as an AUC.

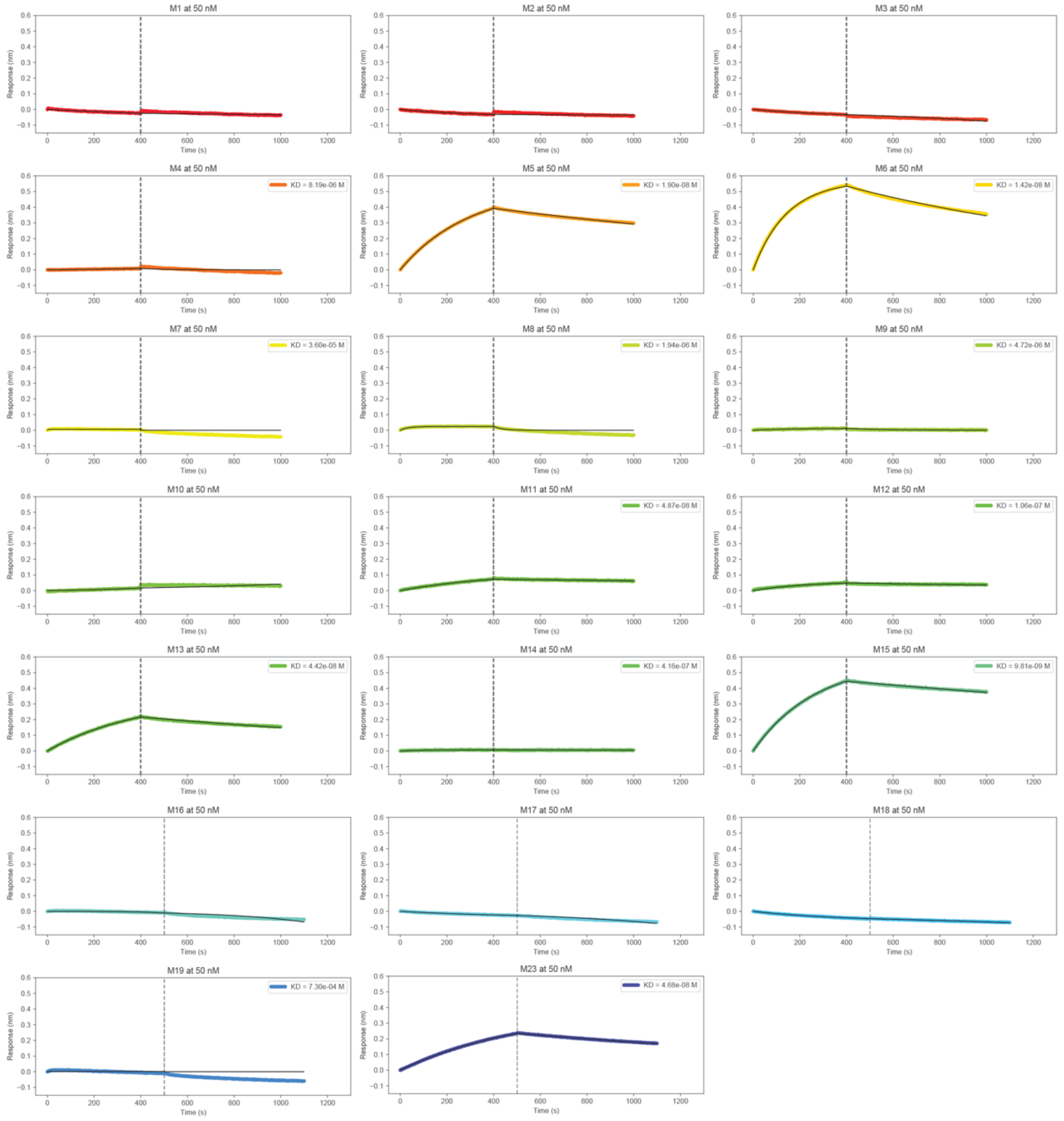

Supplementary Fig. 4 l BLI measured association and dissociation kinetics for the anti-HEL nanobody clones selected from the MACS (M1-M23) library, where nanobody clones were bound at 50 nM to a HEL-biotin loaded streptavidin tip.

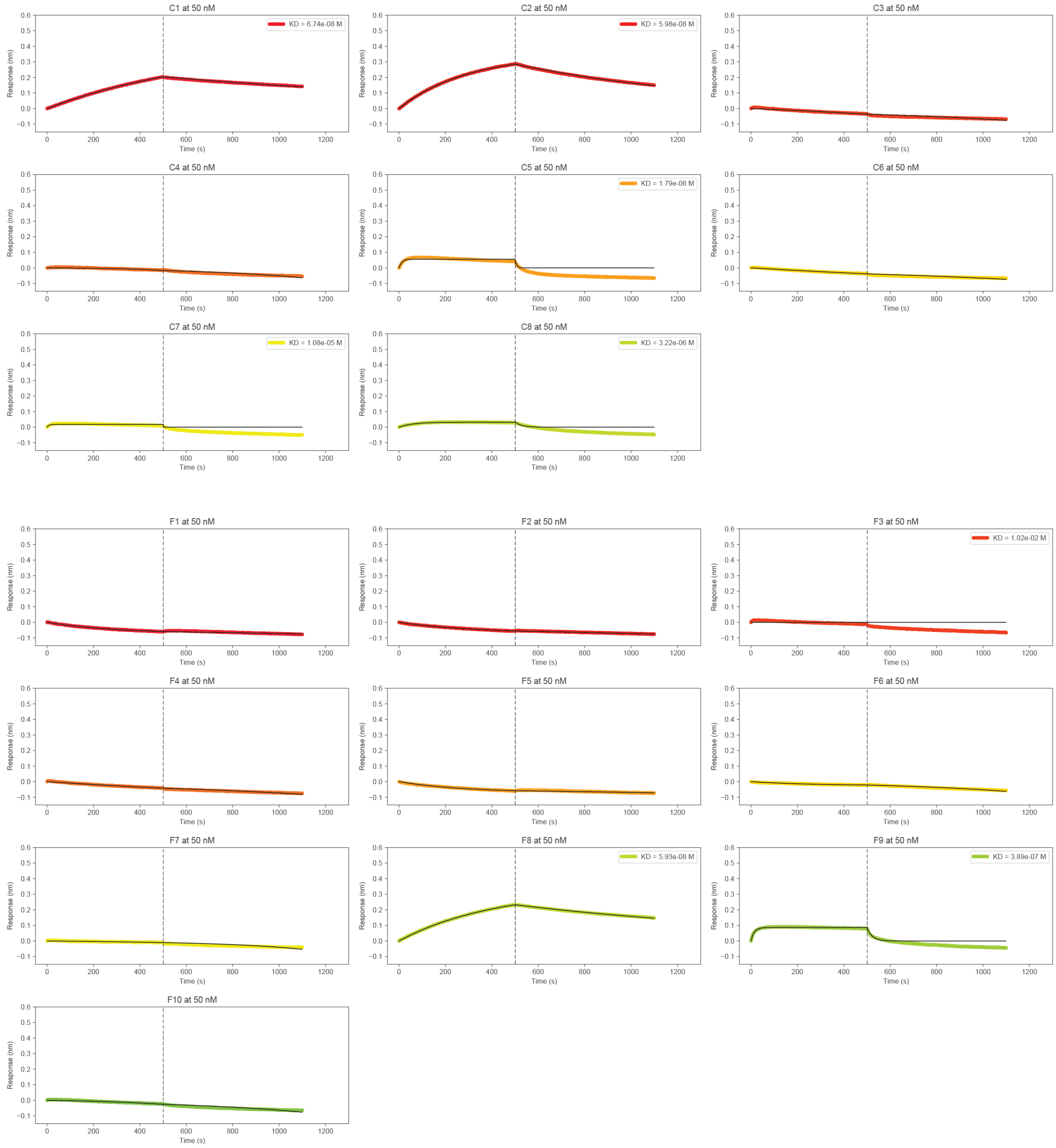

Supplementary Fig. 5 l BLI measured association and dissociation kinetics for the anti-HEL nanobody clones selected from picking 96 colonies (C1-C8) and from the MACS library screen (M1-M10), where nanobody clones were bound at 50 nM to a HEL-biotin loaded streptavidin tip.

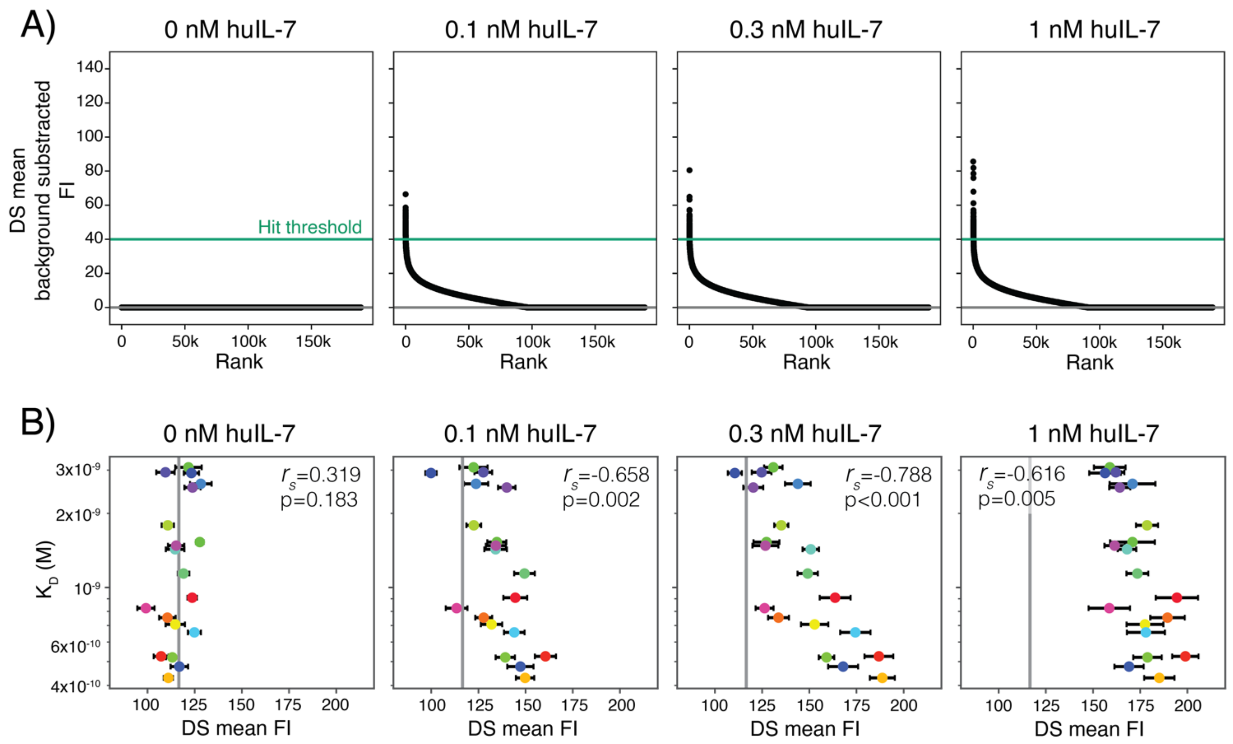

Supplementary Fig. 6 I A) Rank plots of 189k anti-IL7 scFv clones from a deep screening equilibrium binding assay, showing their mean background subtracted fluorescent intensities at 0 nM, 0.1 nM, 0.3 nM and 1 nM huIL-7. A hit threshold at 40 FI units is drawn in green and was empirically determined. The top scoring clones at 1 nM huIL-7 were selected for subsequent conversion to Fab, expression, purification, and characterisation. **B)** Correlation between BLI characterised binding affinities (K_D) and deep screening mean FI at 0 nM, 0.1 nM, 0.3 nM and 1 nM huIL-7. Error bars are standard error of the mean (SEM) and n≥12 technical replicates of a given UMI. The grey vertical line is showing the mean library intensity at each respective concentration. Correlations are shown as Spearman's rank correlation constant (r_s) and p-values determined by a two-tailed test.

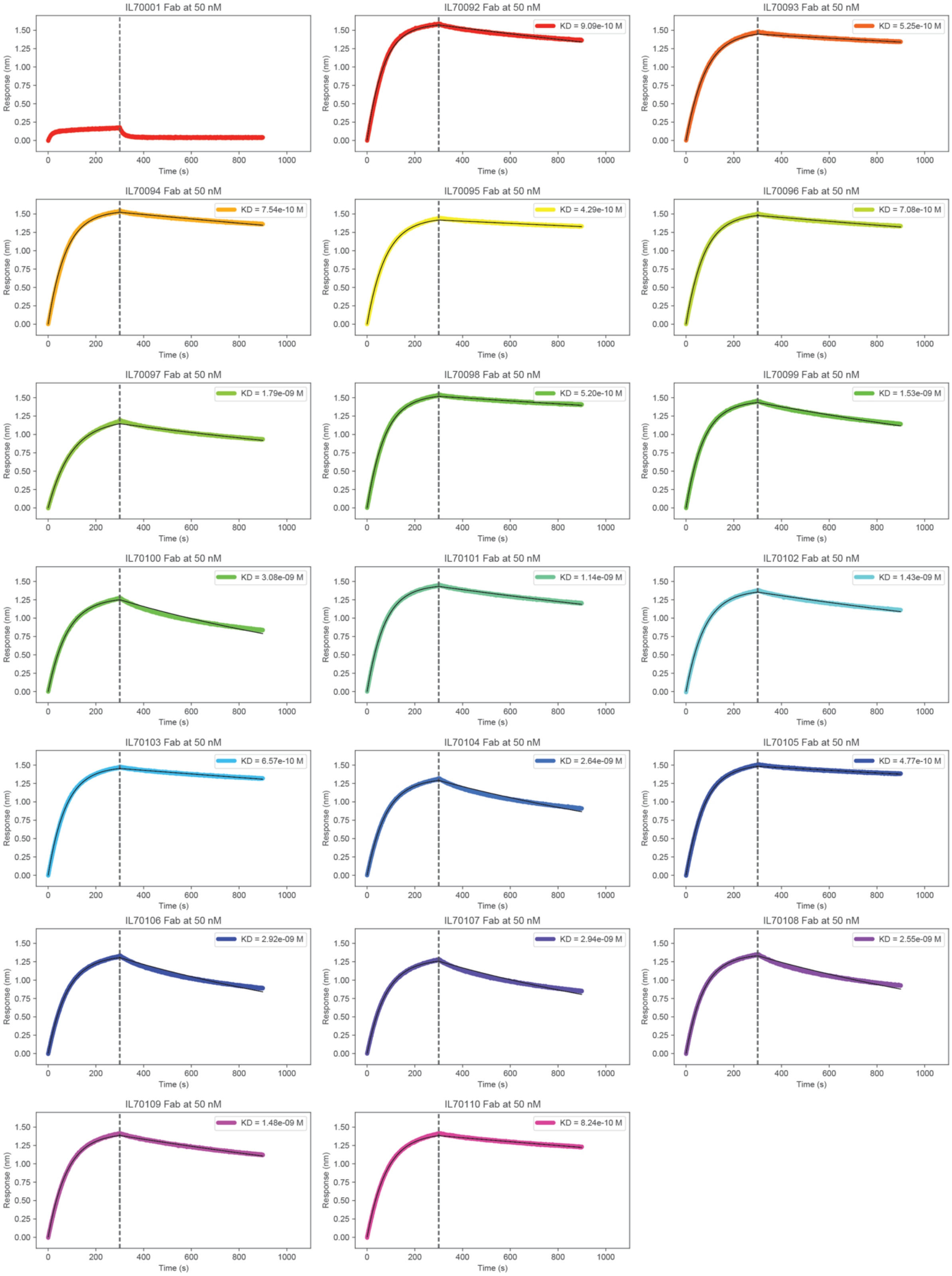

Supplementary Fig. 7 I BLI measured association and dissociation kinetics for the anti-IL7 scFv clones selected for characterisation. Where each clone was converted from scFv to Fab, expressed, purified, and normalised to 50 nM. Fabs were then bound to a streptavidin tip preloaded with huIL7-biotin. A 1:1 model was fit to all clones, except for IL70001.

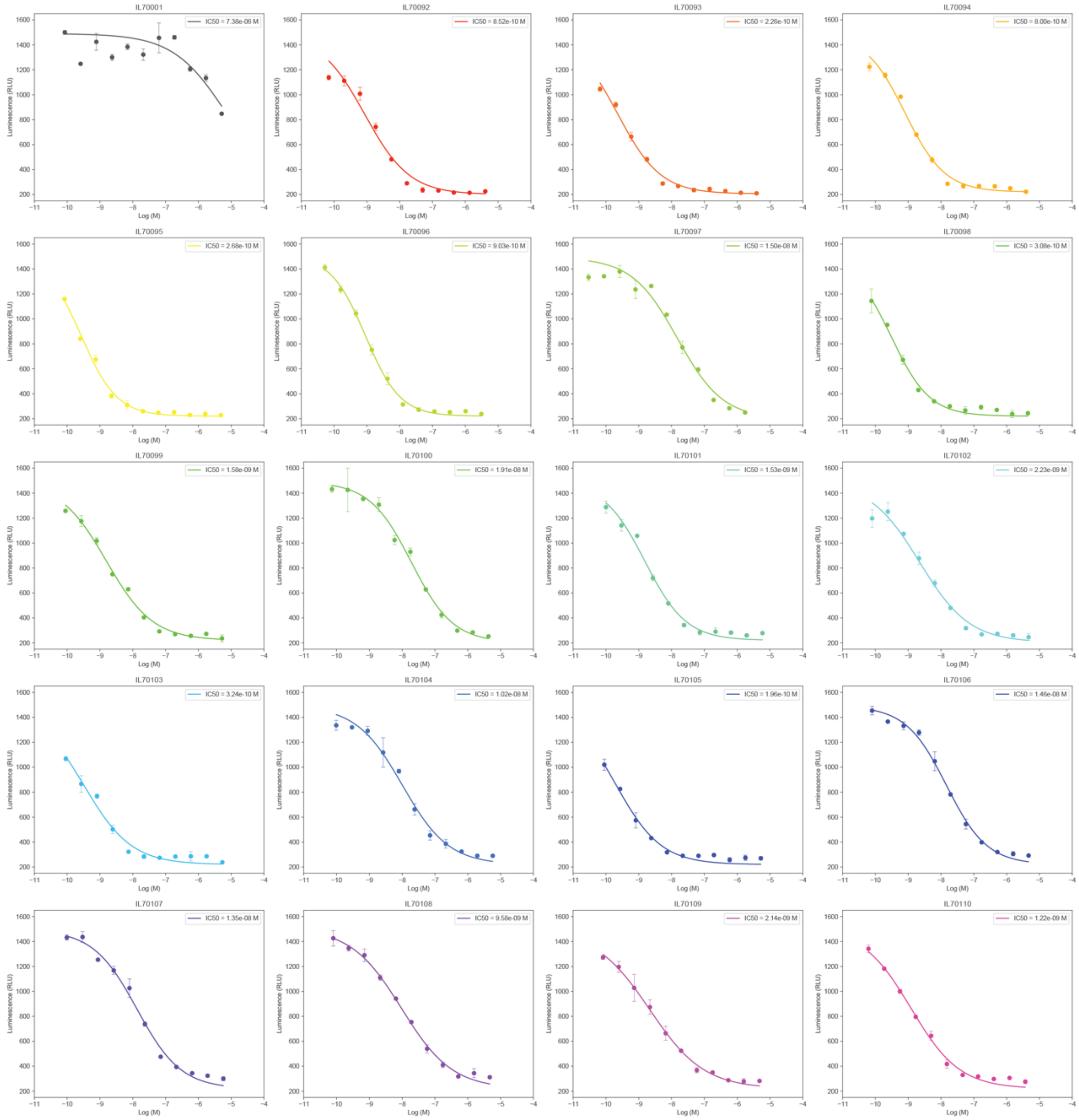

Supplementary Fig. 8 I TF-1 STAT5 IL7 receptor (IL7R) alpha + gamma luciferase inhibition assay, showing IL7R signalling luminescence plotted against the log molar concentration of all characterised clones individually. Error bars are the minimum and maximum observation, n=2 technical replicates.

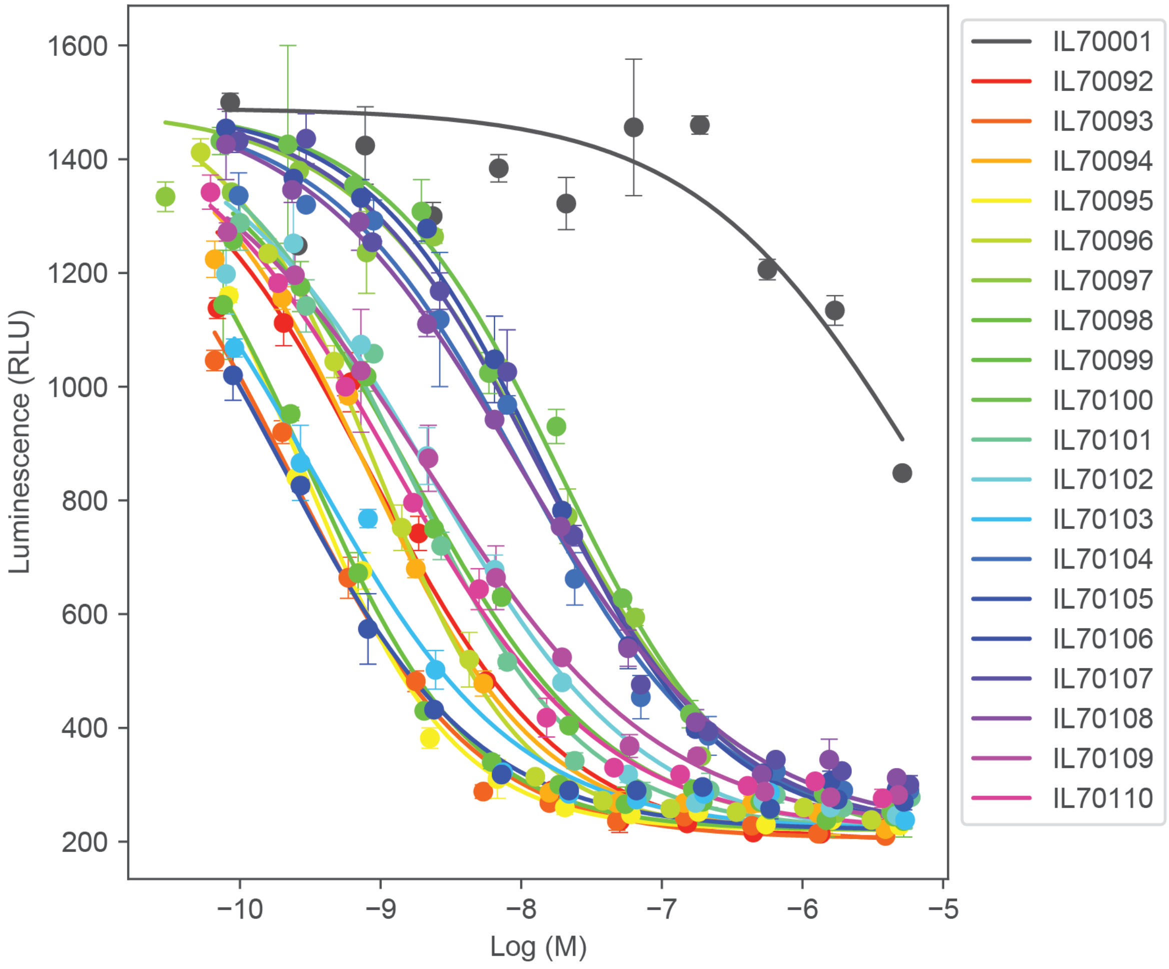

Supplementary Fig. 9 l TF-1 STAT5 IL7 receptor (IL7R) alpha + gamma luciferase inhibition assay, showing IL7R signalling luminescence plotted against the log molar concentration of all characterised clones. Error bars are the minimum and maximum observation, n=2 technical replicates.

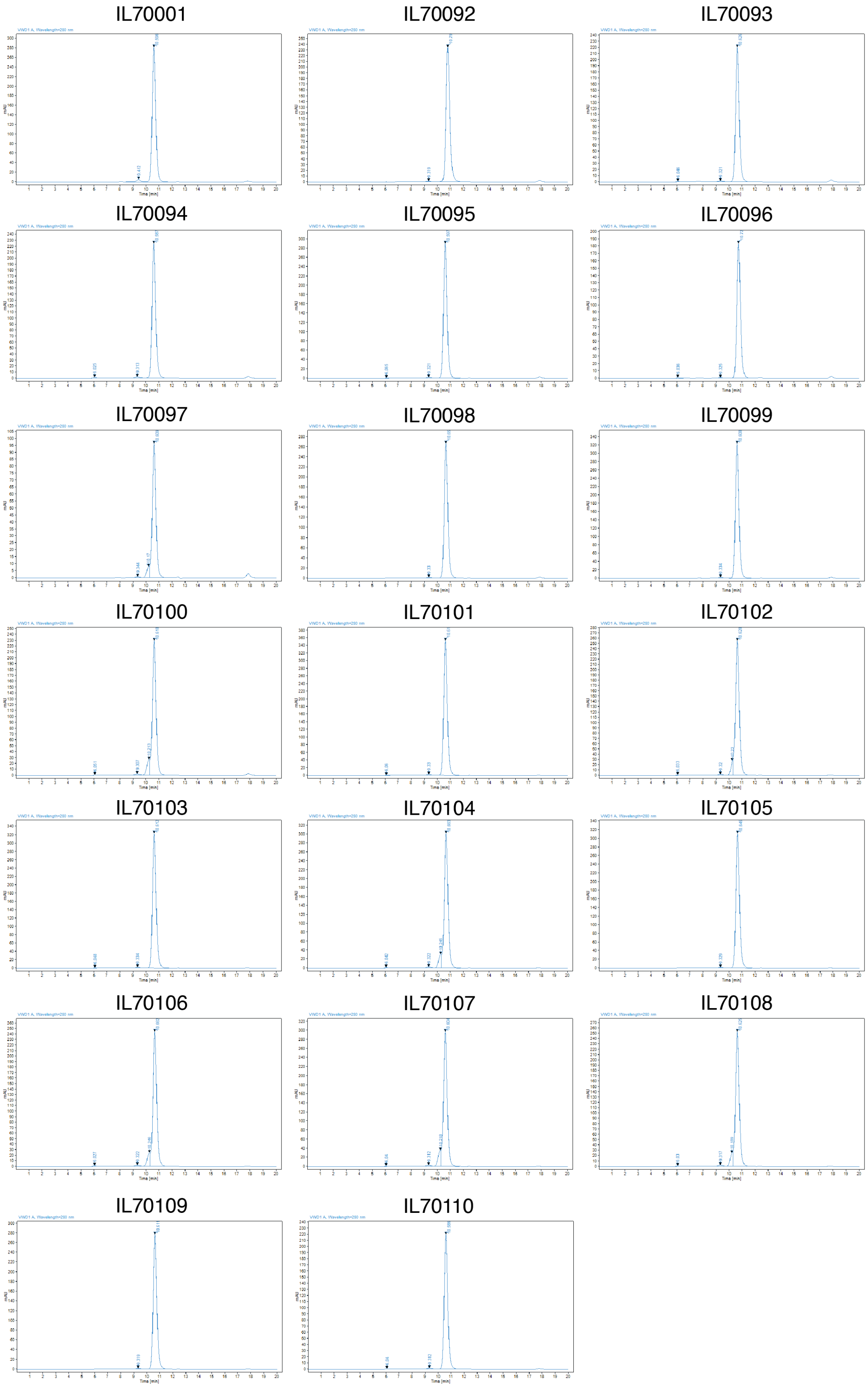

Supplementary Fig. 10 I HP-SEC traces during purification of each anti-IL7 Fab, showing absorbance at 280 nm against retention time in the column.

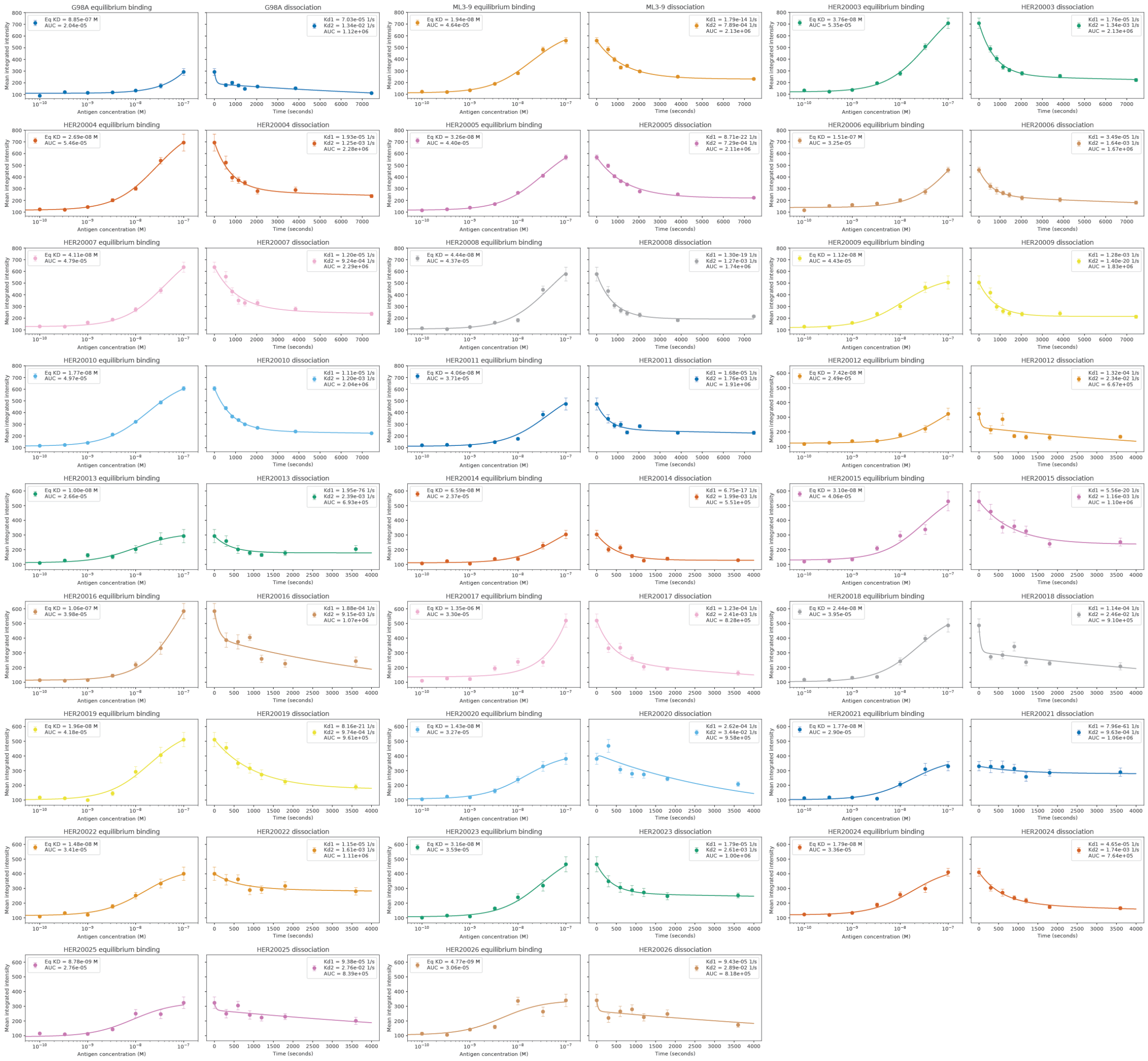

Supplementary Fig. 11 | Deep screening derived equilibrium binding and kinetic dissociation curves for the anti-HER2 scFvs selected for characterisation. Each concentration condition within curve represents at least 12 measurements from either "HER2affmat" (G98A to HER20011) or "HER2 ML vs. Random" (HER20012 to HER20026) deep screening experiments. Error bars are SEM and n≥12 technical replicates of a given UMI. We report an equilibrium KD, area under the curve (AUC) for the equilibrium binding and two dissociation rates for a biphasic dissociation model, as well as an AUC.

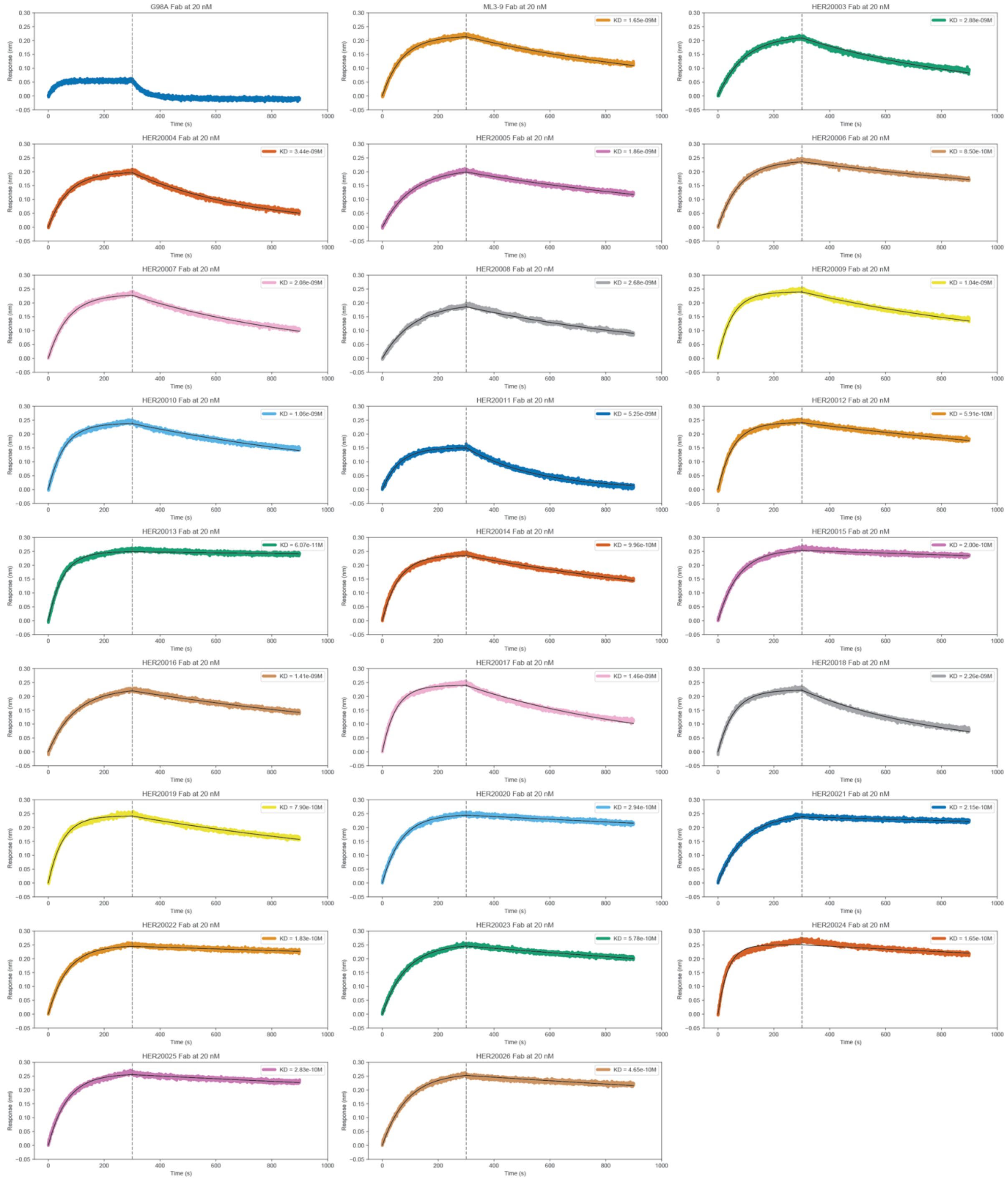

Supplementary Fig. 12 I BLI measured association and dissociation kinetics for the anti-HER2 scFv clones selected for characterisation. Each clone was converted from scFv to Fab, expressed, purified, and normalised to 20 nM. Fabs were then bound to a streptavidin tip preloaded with HER2-biotin. A 1:1 model was fit to all clones, except for G98A.

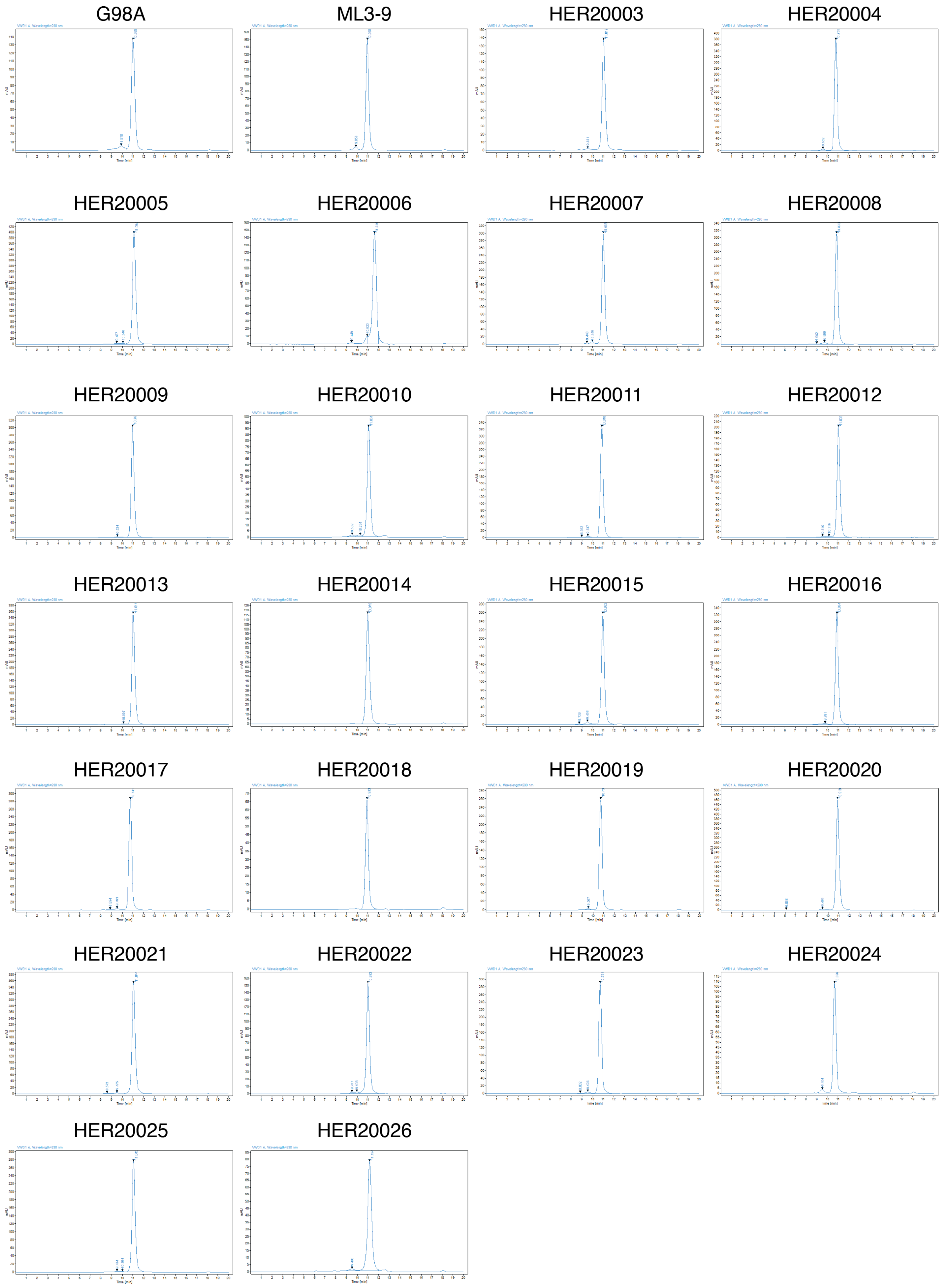

Supplementary Fig. 13 | HP-SEC traces during purification of each anti-HER2 Fab, showing absorbance at 280 nm against retention time in the column.

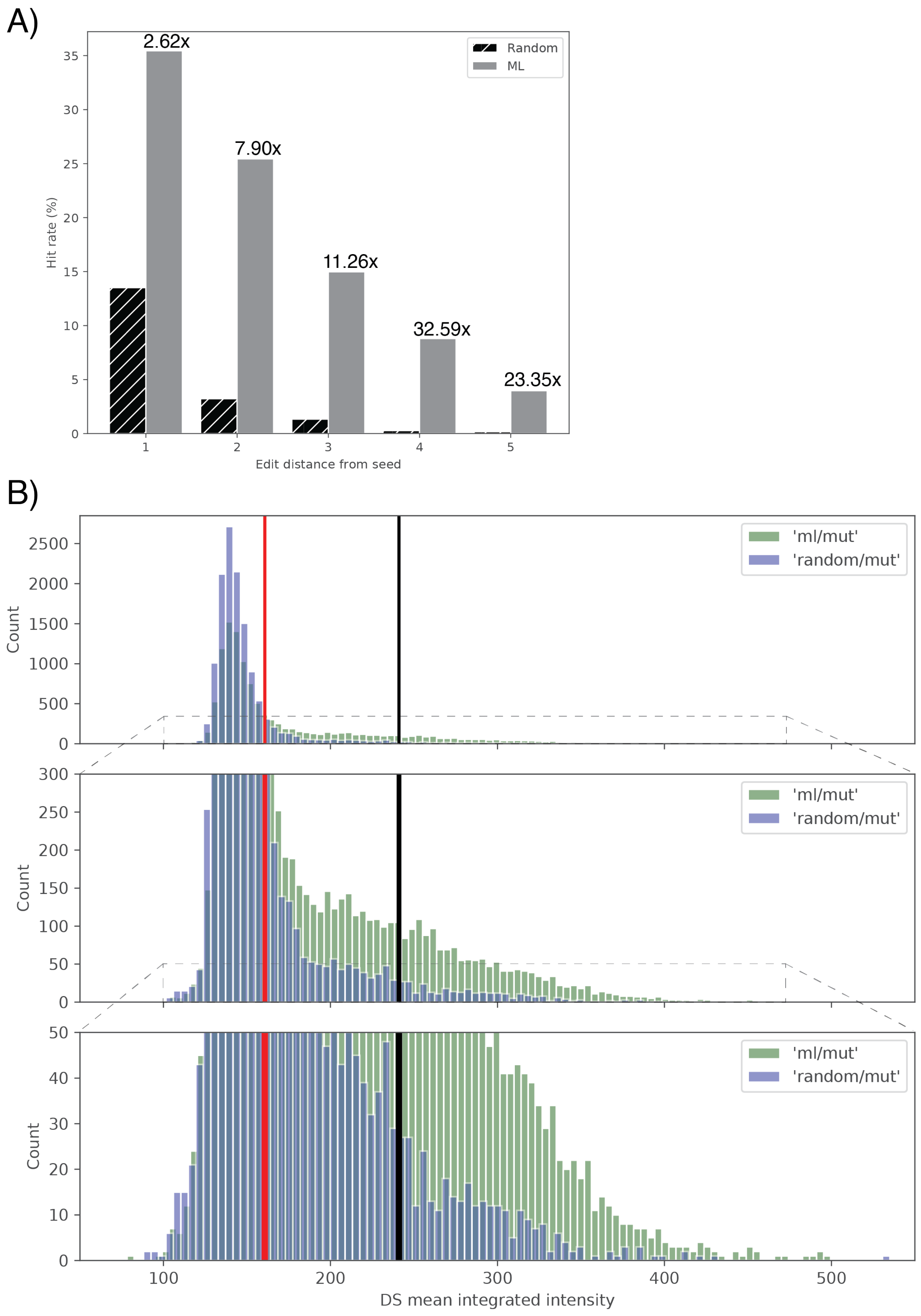

Supplementary Fig. 14 l A) Comparison of hit rate between the random/mut set (black bars with white lines) and the ml/mut set (grey bars) as the edit distance from starting seed sequences is increased from 1 to 5. Numbers above the ml/mut set describe fold improvement over random mutagenesis. Complete numerical values are present in Supplementary Table 4. **B)** Distribution of mean FI values between the 'random/mut' set (blue) and the 'ml/mut' set (green) in the 5-minute wash condition after binding 100 nM HER2. Parental clone G98A is shown as a red vertical line, and the hit threshold (1.5x G98A) is shown a black vertical line.

Supplementary Table 1 | Deep screening construct elements, DNA oligos, DNA oligos for Kruse nanobody library assembly, DNA oligos for internal primer sequencing, anti-HER2 scFv protein sequences, DNA oligos for "HER2Affmat" library assembly.

Deep screening construct elements

Construct element	Sequence
P5 adaptor	AATGATACGGCGACCACCGAGATCTACACTCTTTCCCTACACGACGCTCTTCCGATCT
5p UNS v2	CATTACAAACGACACCCTAAACAAATC
RBS	TATTTTAATAATTAAGGAGGTATATAC
TolAK short linker	TATATGGCTAGTGGTGCCGAATTTGGGTCAGGTGGCCAGAAGCAAGCTGAGGAAGCCGCTGCCAAGGCTGCCGCAGATGCAAAGGCAAAAGCCGAGGCAGACGCTAAAGCTGCGGAAGAGGCTGCGAAAAAGGCAGCAGCCGATGCTAAGAAAAAAGCGGAGGCG
3p UNS v2	TCCTGTTAGACTCCTCAATGCAAGCTG
P7 adaptor	GATCGGAAGAGCACACGTCTGAACTCCAGTCACATCTCGTATGCCGTCTTCTGCTTG

DNA oligos

Oligo name	Sequence
R2_atto647N	/5ATTO647NN//iSpC3/GTGACTGGAGTTCAGACGTGTGCTCTTCCGATC
P7'_surface_hyb	GAACTCCAGTCACATCTCGTATGCCGTCTTCTGCTTG
P5_PCR.fwd	AATGATACGGCGACCACCGA
P7_PCR.rev	CAAGCAGAAGACGGCATACGAGAT

DNA oligos for Kruse nanobody library assembly

Oligo name	Sequence
KF_olap.fwd	ATTAAGGAGGTATATACATGCAGGTGCAGCTGCAGGAAAG
KF_olap.rev	TGACCCAAATTCGGCACCACTAGCCATATAAGCGTAATCTGGAACATCGTATGGG

DNA oligos for internal primer sequencing

Oligo name	Seq cycles	Sequence
Kruse_Nb_CDR1_seq	27	GCCTGAGCTGCGCGGCGAGC
Kruse_Nb_CDR2_seq	42	GCCAGGCGCCGGGCAAAGAACGC

Kruse_Nb_CDR3_seq	57	CCGGAAGATACCGCGGTGTATTATTGCGCG
IL7_scFv_VLCDR1_seq	45	GTCCCCAGGACAGACAGCCAGCATCACC
IL7_scFv_VLCDR3_seq	45	CCGGGACCCAGGCTATGGATGAGGCTGAGTATTAC
HER2_G98A_VH3_seq	63	GCCCTCTGATTCTGCGGTATACTTCTGTGCTCGT

Anti-HER2 scFv protein sequences

**Clone**	**Protein sequence**
G98A	MQVQLVQSGAEVKKPGESLKISCKGSGYSFTSYWIAWVRQMPGKGLEYMGLIYPG DSDTKYSPSFQGQVTISVDKSVSTAYLQWSSLKPSDSAVYFCARHDVAYCSSSNC AKWPEYFQHWGQGTLVTVSSGGGGSGGGGSGGGGSQSVLTQPPSVSAAPGQK VTISCSGSSSNIGNNYVSWYQQLPGTAPKLLIYGHTNRPAGVPDRFSGSKSGTSAS LAISGFRSEDEADYYCASWDYTLSGWVFGGGTKLTVLGDSLEFIASKLAGDSLEFIA SKLADDEGMTGDDSKEAAAKFSTKWWIIDKWRHRPPP
C6.5	MQVQLVQSGAEVKKPGESLKISCKGSGYSFTSYWIAWVRQMPGKGLEYMGLIYPG DSDTKYSPSFQGQVTISVDKSVSTAYLQWSSLKPSDSAVYFCARHDVGYCSSSNC AKWPEYFQHWGQGTLVTVSSGGGGSGGGGSGGGGSQSVLTQPPSVSAAPGQK VTISCSGSSSNIGNNYVSWYQQLPGTAPKLLIYGHTNRPAGVPDRFSGSKSGTSAS LAISGFRSEDEADYYCAAWDDSLSGWVFGGGTKLTVLGDSLEFIASKLAGDSLEFIA SKLADDEGMTGDDSKEAAAKFSTKWWIIDKWRHRPPP
ML3-9	MQVQLVQSGAEVKKPGESLKISCKGSGYSFTSYWIAWVRQMPGKGLEYMGLIYPG DSDTKYSPSFQGQVTISVDKSVSTAYLQWSSLKPSDSAVYFCARHDVGYCSSSNC AKWPEYFQHWGQGTLVTVSSGGGGSGGGGSGGGGSQSVLTQPPSVSAAPGQK VTISCSGSSSNIGNNYVSWYQQLPGTAPKLLIYDHTNRPAGVPDRFSGSKSGTSAS LAISGFRSEDEADYYCASWDYTLSGWVFGGGTKLTVLGDSLEFIASKLAGDSLEFIA SKLADDEGMTGDDSKEAAAKFSTKWWIIDKWRHRPPP
H3B1	MQVQLVQSGAEVKKPGESLKISCKGSGYSFTSYWIAWVRQMPGKGLEYMGLIYPG DSDTKYSPSFQGQVTISVDKSVSTAYLQWSSLKPSDSAVYFCARHDVGYCTDRTC AKWPEYFQHWGQGTLVTVSSGGGGSGGGGSGGGGSQSVLTQPPSVSAAPGQK VTISCSGSSSNIGNNYVSWYQQLPGTAPKLLIYDHTNRPAGVPDRFSGSKSGTSAS LAISGFRSEDEADYYCASWDYTLSGWVFGGGTKLTVLGDSLEFIASKLAGDSLEFIA SKLADDEGMTGDDSKEAAAKFSTKWWIIDKWRHRPPP
B1D2+A1	MQVQLVQSGAEVKKPGESLKISCKGSGYSFTSYWIAWVRQMPGKGLEYMGLIYPG DSDTKYSPSFQGQVTISVDKSVSTAYLQWSSLKPSDSAVYFCARHDVGYCTDRTC AKWPEWLGVWGQGTLVTVSSGGGGSGGGGSGGGGSQSVLTQPPSVSAAPGQK VTISCSGSSSNIGNNYVSWYQQLPGTAPKLLIYDHTNRPAGVPDRFSGSKSGTSAS LAISGFRSEDEADYYCASWDYTLSGWVFGGGTKLTVLGDSLEFIASKLAGDSLEFIA SKLADDEGMTGDDSKEAAAKFSTKWWIIDKWRHRPPP
Herceptin	MEVQLVESGGGLVQPGGSLRLSCAASGFNIKDTYIHWVRQAPGKGLEWVARIYPT NGYTRYADSVKGRFTISADTSKNTAYLQMNSLRAEDTAVYYCSRWGGDGFYAMDV WGQGTLVTVSSGGGGSGGGGSGGGGSTDIQMTQSPSSLSASVGDRVTITCRASQ DVNTAVAWYQQKPGKAPKLLIYSASFLESGVPSRFSGSRSGTDFTLTISSLQPEDFA TYYCQQHYTTPPTFGQGTKVEIK

DNA oligos for “HER2Affmat” library assembly

Oligo name	Sequence
G98A_olap.fwd	ATTAAGGAGGTATATACATGCAGGTACAGCTTGTGCAG
G98A_5p_VH3.rev	ACGAGCACAGAAGTATACCGCA
G98A_3p_VH3.fwd	TGGGGACAAGGGACCCTTGTCAC
G98A_olap.rev	TGACCCAAATTCGGCACCACTAGCCATATACCCAAGCACAGTAAGCTTCGTCC
G98A_VH3_NNS_1	CGGTATACTTCTGTGCTCGTNNSNNSNNSNNSTATTGTTCCAGTAGCAATTGC GCAAAGTGGCCTGAGTATTTCCAACATTGGGGACAAGGGACCCTTGT
G98A_VH3_NNS_2	CGGTATACTTCTGTGCTCGTCATGACGTCNNSNNSNNSNNSAGTAGCAATTGC GCAAAGTGGCCTGAGTATTTCCAACATTGGGGACAAGGGACCCTTGT
G98A_VH3_NNS_3	CGGTATACTTCTGTGCTCGTCATGACGTCGCCTATTGTNNSNNSNNSNNSTGC GCAAAGTGGCCTGAGTATTTCCAACATTGGGGACAAGGGACCCTTGT
G98A_VH3_NNS_4	CGGTATACTTCTGTGCTCGTCATGACGTCGCCTATTGTTCCAGTAGCNNSNNS NNSNNSTGGCCTGAGTATTTCCAACATTGGGGACAAGGGACCCTTGT
G98A_VH3_NNS_5	CGGTATACTTCTGTGCTCGTCATGACGTCGCCTATTGTTCCAGTAGCAATTGC GCANNSNNSNNSNNSTATTTCCAACATTGGGGACAAGGGACCCTTGT
G98A_VH3_NNS_6	CGGTATACTTCTGTGCTCGTCATGACGTCGCCTATTGTTCCAGTAGCAATTGC GCAAAGTGGCCTNNSNNSNNSNNSCATTGGGGACAAGGGACCCTTGT

Supplementary Table 2 I BERT-DS (pre-trained in 2021) fine-tuned train:test confusion matrix.

Train	Count	TP	FP	TN	FN
Non-hit	209,414	206,564	277	988	2,850
Low-hit	1,168	839	2,882	206,629	329
High-hit	97	53	64	210,518	44
**Test**	**Count**	**TP**	**FP**	**TN**	**FN**
Non-hit	23,279	22,984	31	99	295
Low-hit	116	82	301	22,992	34
High-hit	14	6	5	23,390	8

Supplementary Table 3 I BERT-DS (pre-trained in 2021) fine-tuning performance.

Train	Precision*	Recall**	F1 score***
Non-hit	0.999	0.986	0.992
Low-hit	0.225	0.718	0.343
High-hit	0.453	0.546	0.495
**Test**	**Precision***	**Recall****	**F1 score*****
Non-hit	0.999	0.987	0.993
Low-hit	0.214	0.707	0.329
High-hit	0.545	0.429	0.480

*Precision is defined as: TP/(TP+FP)
**Recall is defined as: TP/(TP+FN)
***F1 score is defined as the harmonic mean of precision and recall.

Supplementary Table 4 I ML vs. Random selected clones; hit* performance.

Edit distance	Random total	Random hits	Random hit rate (%)	ML total	ML hits	ML hit rate (%)	ML fold improvement
**1**	1,140	154	13.51	220	78	35.45	2.62
**2**	2,981	96	3.22	2,932	746	25.44	7.90
**3**	3,000	40	1.33	2,984	447	14.98	11.26
**4**	3,000	8	0.27	3,000	264	8.80	32.59
**5**	3,000	5	0.17	3,000	119	3.97	23.35
**Total**	13,121	303	2.31	12,136	1,654	13.62	5.90
			**Total****	11,916	1,576	13.23	5.73

*Hits are defined as clones with fluorescent intensities >= 1.5x G98A in the 5-minute wash condition.
**This total excludes the single point mutations from the ML set.

Supplementary Table 5 | Ablation study: BERT-DS (with pre-training) fine-tuned performance.

**Train**	**Precision***	**Recall****	**F1 score*****
Non-hit	1.000	0.994	0.997
Low-hit	0.507	0.987	0.670
High-hit	0.794	1.000	0.885
**Test**	**Precision***	**Recall****	**F1 score*****
Non-hit	0.998	0.991	0.994
Low-hit	0.296	0.588	0.394
High-hit	0.429	0.409	0.419

*Precision is defined as: TP/(TP+FP)
**Recall is defined as: TP/(TP+FN)
***F1 score is defined as the harmonic mean of precision and recall.

Supplementary Table 6 | Ablation study: BERT-DS (with pre-training) fine-tuned performance with a soft classification target.

**Train**	**Precision***	**Recall****	**F1 score*****
Non-hit	1.000	0.992	0.996
Low-hit	0.441	0.981	0.609
High-hit	0.729	0.963	0.830
**Test**	**Precision***	**Recall****	**F1 score*****
Non-hit	0.998	0.991	0.994
Low-hit	0.307	0.616	0.410
High-hit	0.233	0.455	0.308

*Precision is defined as: TP/(TP+FP)
**Recall is defined as: TP/(TP+FN)
***F1 score is defined as the harmonic mean of precision and recall.

Supplementary Table 7 | Ablation study: BERT-DS (random initialisation) fine-tuned performance.

**Train**	**Precision***	**Recall****	**F1 score*****
Non-hit	1.000	0.996	0.998
Low-hit	0.637	0.994	0.777
High-hit	0.771	1.000	0.871
**Test**	**Precision***	**Recall****	**F1 score*****
Non-hit	0.997	0.993	0.995
Low-hit	0.343	0.555	0.424
High-hit	0.333	0.455	0.385

*Precision is defined as: TP/(TP+FP)
**Recall is defined as: TP/(TP+FN)
***F1 score is defined as the harmonic mean of precision and recall.

Supplementary Table 8 l Ablation study: BERT-DS (random initialisation) fine-tuned performance with a soft classification target.

**Train**	**Precision***	**Recall****	**F1 score*****
Non-hit	1.000	0.990	0.995
Low-hit	0.355	0.939	0.515
High-hit	0.503	0.951	0.658
**Test**	**Precision***	**Recall****	**F1 score*****
Non-hit	0.998	0.987	0.993
Low-hit	0.237	0.600	0.340
High-hit	0.200	0.455	0.278

*Precision is defined as: TP/(TP+FP)
**Recall is defined as: TP/(TP+FN)
***F1 score is defined as the harmonic mean of precision and recall.

Supplementary Table 9 l Multi-layered perceptron (MLP) train:test precision, recall and F1 score.

**Train**	**Precision***	**Recall****	**F1 score*****
Non-hit	1.000	0.998	0.999
Low-hit	0.745	0.974	0.844
High-hit	0.868	0.975	0.919
**Test**	**Precision***	**Recall****	**F1 score*****
Non-hit	0.997	0.997	0.997
Low-hit	0.457	0.478	0.467
High-hit	0.211	0.182	0.195

*Precision is defined as: TP/(TP+FP)
**Recall is defined as: TP/(TP+FN)
***F1 score is defined as the harmonic mean of precision and recall.

Supplementary Table 10 l Multi-layered perceptron (MLP) with a soft classification target, train:test precision, recall and F1 score.

**Train**	**Precision***	**Recall****	**F1 score*****
Non-hit	1.000	0.995	0.997
Low-hit	0.527	0.966	0.682
High-hit	0.841	0.914	0.876
**Test**	**Precision***	**Recall****	**F1 score*****
Non-hit	0.998	0.994	0.996
Low-hit	0.384	0.580	0.462
High-hit	0.286	0.273	0.280

*Precision is defined as: TP/(TP+FP)
**Recall is defined as: TP/(TP+FN)
***F1 score is defined as the harmonic mean of precision and recall.

Supplementary Table 11 l Logistic regression train:test precision, recall and F1 score.

Train	Precision*	Recall**	F1 score***
Non-hit	1.00	1.00	1.00
Low-hit	0.66	0.16	0.26
High-hit	0.82	0.09	0.16
**Test**	**Precision***	**Recall****	**F1 score*****
Non-hit	0.99	1.00	1.00
Low-hit	0.70	0.19	0.29
High-hit	0.00	0.00	0.00

*Precision is defined as: TP/(TP+FP)
**Recall is defined as: TP/(TP+FN)
***F1 score is defined as the harmonic mean of precision and recall.

Supplementary Table 12 l Linear Support Vector Machine train:test precision, recall and F1 score.

Train	Precision*	Recall**	F1 score***
Non-hit	0.99	1.00	1.00
Low-hit	0.50	0.00	0.00
High-hit	0.60	0.09	0.16
**Test**	**Precision***	**Recall****	**F1 score*****
Non-hit	0.99	1.00	1.00
Low-hit	0.00	0.00	0.00
High-hit	0.50	0.17	0.25

*Precision is defined as: TP/(TP+FP)
**Recall is defined as: TP/(TP+FN)
***F1 score is defined as the harmonic mean of precision and recall.

Supplementary Table 13 l Random Forest classifier train:test precision, recall and F1 score.

Train	Precision*	Recall**	F1 score***
Non-hit	1.00	1.00	1.00
Low-hit	1.00	1.00	1.00
High-hit	1.00	1.00	1.00
**Test**	**Precision***	**Recall****	**F1 score*****
Non-hit	0.99	1.00	1.00
Low-hit	0.55	0.04	0.07
High-hit	1.00	0.08	0.15

*Precision is defined as: TP/(TP+FP)
**Recall is defined as: TP/(TP+FN)
***F1 score is defined as the harmonic mean of precision and recall.

Supplementary Table 14 I F1 scores from all models, including BERT-DS as described in the main text and those described in the ablation study.

	**BERT-DS (2021)**	**BERT-DS pre-trained**	**BERT-DS pre-trained soft target**	**BERT-DS random initialisation**	**BERT-DS random initialisation soft target**	**MLP**	**MLP soft target**	**Logistic regression**	**Linear SVM**	**Random forest**
**Train**										
Non-hit	0.99	1.00	1.00	1.00	1.00	1.00	1.00	1.00	1.00	1.00
Low-hit	0.34	0.67	0.61	0.78	0.52	0.84	0.68	0.26	0.00	1.00
High-hit	0.50	0.89	0.83	0.87	0.66	0.92	0.88	0.16	0.16	1.00
**Test**										
Non-hit	0.99	0.99	0.99	1.00	0.99	1.00	1.00	1.00	1.00	1.00
Low-hit	0.33	0.39	0.41	0.42	0.34	0.47	0.46	0.29	0.00	0.07
High-hit	0.48	0.42	0.31	0.39	0.28	0.20	0.28	0.00	0.25	0.15
