# Supplementary material for: Rapid discovery of high-affinity antibodies via massively parallel sequencing, ribosome display and affinity screening
Source: Nat Biomed Eng. 2023 Oct 9;8(3):214–32. doi: 10.1038/s41551-023-01093-3 (PMC10963267; doi:10.1038/s41551-023-01093-3)
Supplement: Supplementary file 2 — Reporting Summary [file 41551_2023_1093_MOESM2_ESM.pdf]

nature portfolio

Corresponding author(s): Philipp Holliger
Last updated by author(s): Jul 26, 2023

Reporting Summary

Nature Portfolio wishes to improve the reproducibility of the work that we publish. This form provides structure for consistency and transparency in reporting. For further information on Nature Portfolio policies, see our Editorial Policies and the Editorial Policy Checklist.

Statistics

For all statistical analyses, confirm that the following items are present in the figure legend, table legend, main text, or Methods section.

n/a	Confirmed	
☐	☒	The exact sample size (*n*) for each experimental group/condition, given as a discrete number and unit of measurement
☐	☒	A statement on whether measurements were taken from distinct samples or whether the same sample was measured repeatedly
☒	☐	The statistical test(s) used AND whether they are one- or two-sided<br>*Only common tests should be described solely by name; describe more complex techniques in the Methods section.*
☒	☐	A description of all covariates tested
☒	☐	A description of any assumptions or corrections, such as tests of normality and adjustment for multiple comparisons
☐	☒	A full description of the statistical parameters including central tendency (e.g. means) or other basic estimates (e.g. regression coefficient) AND variation (e.g. standard deviation) or associated estimates of uncertainty (e.g. confidence intervals)
☒	☐	For null hypothesis testing, the test statistic (e.g. *F*, *t*, *r*) with confidence intervals, effect sizes, degrees of freedom and *P* value noted<br>*Give P values as exact values whenever suitable.*
☒	☐	For Bayesian analysis, information on the choice of priors and Markov chain Monte Carlo settings
☒	☐	For hierarchical and complex designs, identification of the appropriate level for tests and full reporting of outcomes
☐	☒	Estimates of effect sizes (e.g. Cohen's *d*, Pearson's *r*), indicating how they were calculated

Our web collection on statistics for biologists contains articles on many of the points above.

Software and code

Policy information about availability of computer code

Data collection	HiSeq Control software (HCS v. 2.2.68, Illumina).<br>Archimedes Test software (ATS v. 3.8.317.0, Illumina).<br>Octet Data Acquisition software (v. 11.0, ForteBio).
Data analysis	Data were analysed using custom software, available at https://github.com/holliger-lab/DeepScreening

For manuscripts utilizing custom algorithms or software that are central to the research but not yet described in published literature, software must be made available to editors and reviewers. We strongly encourage code deposition in a community repository (e.g. GitHub). See the Nature Portfolio guidelines for submitting code & software for further information.

Data

Policy information about availability of data

All manuscripts must include a data availability statement. This statement should provide the following information, where applicable:

- Accession codes, unique identifiers, or web links for publicly available datasets
- A description of any restrictions on data availability
- For clinical datasets or third party data, please ensure that the statement adheres to our policy

The main data supporting the results in this study are available in summarized form from Zenodo at https://doi.org/10.5281/zenodo.8241732, under a CC-BY-NC-

ND licence. The raw datasets generated during the study are too large to be publicly shared, yet these and other raw data are available from the corresponding author on reasonable request.

Human research participants

Policy information about studies involving human research participants and Sex and Gender in Research.

Reporting on sex and gender	The study did not involve human participants.
Population characteristics	—
Recruitment	—
Ethics oversight	—

Note that full information on the approval of the study protocol must also be provided in the manuscript.

Field-specific reporting

Please select the one below that is the best fit for your research. If you are not sure, read the appropriate sections before making your selection.

☒ Life sciences ☐ Behavioural & social sciences ☐ Ecological, evolutionary & environmental sciences

For a reference copy of the document with all sections, see nature.com/documents/nr-reporting-summary-flat.pdf

Life sciences study design

All studies must disclose on these points even when the disclosure is negative.

Sample size	Sample sizes for the deep-screening experiments were initially determined by Illumina sequencing and refined via UMI replicate filtering, median absolute deviation outlier rejection, and filters on whether data were observed during the binding assay.
Data exclusions	In processing the deep-screening data, measurements were grouped by UMI, and outliers removed by median absolute deviation of 2 standard deviations from the sample median.
Replication	The deep-screening experiments were not replicated, although replication studies have been performed outside of this work. Within a given deep-screening experiment, we require that each UMI has at least 12 replicate measurements at different locations on the flow-cell surface. Otherwise a UMI is excluded from further analysis.<br><br>The BLI experiments were not replicated.<br><br>TF-1 STAT-5 IL7 receptor-signalling assays were performed in duplicate for each anti-IL7 Fab that was produced.
Randomization	No randomization outside of random mutagenesis (experimental/in silico) was performed or required.
Blinding	Blinding was not applicable to this study, because data acquisition and quantification were performed by machines.

Reporting for specific materials, systems and methods

We require information from authors about some types of materials, experimental systems and methods used in many studies. Here, indicate whether each material, system or method listed is relevant to your study. If you are not sure if a list item applies to your research, read the appropriate section before selecting a response.

Materials & experimental systems

n/a	Involved in the study	
☐	☒	Antibodies
☒	☐	Eukaryotic cell lines
☒	☐	Palaeontology and archaeology
☒	☐	Animals and other organisms
☒	☐	Clinical data
☒	☐	Dual use research of concern

Methods

n/a	Involved in the study	
☒	☐	ChIP-seq
☐	☒	Flow cytometry
☒	☐	MRI-based neuroimaging

Antibodies

Antibodies used	FITC labelled anti-HA antibody (GG8-1F3.3.1, Miltenyi Biotech).
Validation	The manufacturer states that 293HEK cells transiently transfected with HA-tagged CD4 were stained intracellularly with Anti-HA-FITC and CD4-PE, and analysed by flow cytometry to control gene-of-interest and MACSelect surface-marker expression.<br>The manufacturer shows a flow-cytometry plot that indicates the validation of the product.<br>https://www.miltenyibiotec.com/GB-en/products/ha-antibody-gg8-1f3-3-1.html#fitc:30-tests-in-60-ul

Flow Cytometry

Plots

Confirm that:

☒ The axis labels state the marker and fluorochrome used (e.g. CD4-FITC).

☒ The axis scales are clearly visible. Include numbers along axes only for bottom left plot of group (a 'group' is an analysis of identical markers).

☒ All plots are contour plots with outliers or pseudocolor plots.

☒ A numerical value for number of cells or percentage (with statistics) is provided.

Methodology

Sample preparation	The nanobody yeast-display library was acquired from the Kruse laboratory as a frozen stock of >2.5x10^9 cells (EF0014-FP, Kerafast). To prepare the naïve library for the first round of selection, one aliquot was thawed at 30°C and used to inoculate 1 L of Yglc4.5 –Trp supplemented with 2% galactose. The culture was then grown for 72 hours at 24°C. Expression was confirmed by flow cytometry with a FITC-labelled anti-HA antibody (GG8-1F3.3.1, Miltenyi Biotech) prior to the first round of selection. Round 3 FACS was conducted by incubating cells with 200 nM HEL-biotin for one hour at 4°C, pelleted and resuspended in fresh PBS-T-BSA and combined with 100 µg of Neutravidin-PE (A2660, ThermoFisher Scientific) and a 1:1000 dilution of the anti-HA-FITC (GG8-1F3.3.1, Miltenyi Biotech) antibody for 15 minutes before being sorted.
Instrument	Synergy 3 cell sorter (Sony Biotechnology).
Software	SY3200 software v2.0 (Sony Biotechnology).
Cell population abundance	50,135 cells with dual labelled (FITC/PE).
Gating strategy	Cells were sorted by gating for dual labelled (FITC/PE) events, yielding 50,135 cells.

☒ Tick this box to confirm that a figure exemplifying the gating strategy is provided in the Supplementary Information.
